# The Case for Low-Friction High-Adherence Passive Sleep-Tracking Devices in Longitudinal Monitoring of Neurodegenerative Disease

**DOI:** 10.3390/s26216670

**Published:** 2026-10-22

**Authors:** Angelika Papanicolaou, Graham Jones

**Affiliations:** 1 Tufts University Medical Center, Boston, MA 02111, USA; 2 Clinical Innovation, Novartis Pharmaceuticals, Cambridge, MA 02139, USA

**Keywords:** smart ring, remote monitoring, Parkinson’s disease, passive

## Abstract

Digital health technologies are playing an increasingly important role in patient monitoring, able to capture longitudinal data to supplement scheduled episodic evaluations conducted by clinicians. Rapid advances in sensors and data processing algorithms have resulted in some highly sophisticated systems capable of monitoring and tracking numerous symptoms with high levels of sensitivity and specificity. These approaches, however, often require active engagement with the patient, potentially increasing burden in a clinical trial and often resulting in diminishing adherence over time. In the case of slow-progressing neurodegenerative diseases, this may become an obstacle, as often very subtle changes in symptoms are typically monitored over a multi-year period. Accordingly, there is evident interest in passive monitoring solutions which have minimal burden on patients. Smart ring technologies are one such solution, with appealing form factors, low engagement requirements and increasing intervals before device recharging is required. While newer devices are capable of tracking sleep function with increasing levels of accuracy, they are unable to offer the precision afforded by gold standard sleep analysis such as electroencephalography (EEG), polysomnography (PSG) and sleep laboratory protocols. Prompted by a recent study which highlighted the reduced accuracy of consumer-based sleep devices compared with conventional approaches, we posit that the benefits of such devices may offset the constraints of measurement accuracy. Furthermore, any accuracy–adherence trade-offs would be compensated by long-term statistical benefits derived from detecting within-person changes and reducing measurement noise through enhanced sampling frequencies. In this perspective, we highlight their potential utility in tracking Parkinson’s disease patients longitudinally, where compromised sleep architectures are associated with multiple symptoms and continuous monitoring could offer myriad patient benefits.

## 1. Background and Introduction

Parkinson’s disease (PD) is a progressive neurodegenerative condition associated with the gradual decline in motor and cognitive functions coupled with an array of symptoms impacting quality of life [1]. Following diagnosis (current estimates suggest over 10M cases globally and over 1M in the USA alone), patients are assessed and monitored according to standardized scales such as the MDS-UPDRS and Hoehn–Yahr [2,3]. With many patients entering the prodromal phases in their early forties, assessments are typically episodic and conducted annually or semi-annually in clinical settings over the following decades. Although a number of promising clinical trials are underway, no disease-modifying therapies are currently approved [4], but symptomatic remedies are available to attenuate tremor-related symptoms (dopamine analogs) [5], and there is evidence to suggest lifestyle factors (exercise regimens, dietary interventions) may hold promise to slow progression of the disease [6]. Of the myriad symptoms experienced by patients, sleep abnormalities exert a commanding influence and are exacerbated by the disease itself as the progressive neurodegenerative processes directly attack the brainstem and hypothalamus, responsible for regulating sleep–wake cycles and the internal body clock [7]. Some examples of observed symptoms include:

Physical

- Impaired motor control: Compromised rest significantly increases fatigue, exacerbating physical tremors, rigidity, and bradykinesia (slowness of movement) [8].
- Increase in pain: Chronic lack of sleep lowers pain thresholds, rendering muscle cramping (dystonia) and joint stiffness to feel increasingly severe [9].
- Balance and falls: Chronic daytime sleepiness and fatigue decrease patient alertness, impairing balance and increasing risk of falling [10].

Cognitive and Psychological

- Mental fog: Compromised sleep severely impairs memory, executive function and concentration, which can accentuate dementia-like symptoms [11].
- Mood disorders: Fragmented sleep and insomnia are related to increased levels of anxiety, depression, and repetitive thoughts [12].

The disease itself actively contributes to limiting or preventing restorative processes through a number of mechanisms including nocturnal akinesia (which restricts movement or turning over due to stiffness and rigidity, resulting in frequent awakenings) [13], REM sleep behavior disorder (RBD) associated with vivid, often-traumatic episodes which prevent deep sleep [14], and autonomic dysfunction including nocturia (frequent nocturnal bathroom use, disrupting sleep cycles) [15]. There is also the potential for medication side-effects associated with dopaminergic (symptomatic) agents including sudden daytime sleep attacks and nocturnal insomnia [16]. Accordingly, monitoring and managing sleep disruptions and associated symptoms represents an important component for maintaining and improving patient quality of life (Table 1).

## 2. The Rise of Consumer-Grade Sleep Trackers

The rapid development of wearable digital health technologies promises to revolutionize health and disease monitoring and is starting to gain traction among the regulatory authorities [17]. In the area of PD alone, numerous devices have been developed to capture and track gait and tremor-related motor functions [18,19], including custom-designed ring-based technologies [20,21,22]. As sensors and algorithms which analyze captured data continue to improve, we are approaching a nexus where the sophistication and sensitivity of the devices may need to be balanced by convenience and ease of adoption and use. In the case of PD, this is critical, as the case can be argued that having a device with little or low burden yet with minimal features may be better suited for long-term tracking (over multiple decades) to allow true longitudinal monitoring to be achieved. In this case, a smart ring offers a potentially ideal compromise, with minimal form factor, ability to track rudimentary signals related to sleep function, and potential for direct-to-cloud data upload to minimize patient engagement. In this scenario, the only user-initiated function would be device charging periodically, and even this may be circumventable if motion or solar-provisioned charging were introduced [23]. These considerations were brought to public attention through a recent study which compared sleep tracking obtained by wrist-worn and ring-based wearables to gold standard polysomnogram-based analysis in an academic sleep laboratory [24]. The wearables all measured heart rate effectively, but over-estimated the percentage of deep sleep. Notably, however, the authors suggested that overall sleep duration and detection of REM sleep provided reasonable approximations [24]. This is significant given that the optical PPG sensors on the ring device were not refined for tracking sleep anomalies, and suggest that improvements may be possible given the positive correlations with heart rate obtained. For example, in the case of PD, it is likely that a patient experiencing a hallucination event would register a characteristic cardiac signal [25] and, coupled with long-term estimation of sleep duration and REM cycling, could paint a useful picture of background sleep and unique episodic events to their attending clinician. It is also reasonable to expect that advances in sensor technology and algorithm optimization will help refine the clinical utility of smart ring-derived sleep measurements. Accuracy for sleep–wake detection has been reported in the 94% range in clinical studies with even higher accuracies attainable when combined with additional signals [26]. Moreover, head-to-head comparison with PSG-based approaches have confirmed good correlation with several key measures including sleep onset latency, total sleep time, and wake after sleep onset providing momentum for future development [27]. Although there currently exists a marked gap in the validation-readiness of smart-ring approaches compared to PSG, controlled studies in healthy subjects continue to help define and prioritize parameters to be optimized, and the ubiquity of the devices globally will likely help accelerate this goal [28].

## 3. Why Patient Adherence Is Important in PD

For any useful long-term monitoring deployment, any digital health technology needs to have high adherence. Studies show that even in healthy individuals, sustained use of apps and technologies for health tracking wanes over time [29]. In the case of neurodegenerative diseases, this situation is exacerbated, with numerous clinical studies reporting a substantial lack of adherence over time, which is worsened when patient burden is higher such as completing digital PROs [30], performing tasks on digital devices [31], and even the burden of device charging [32]. As a case in point, among PD patients, even adherence to symptom-alleviating medications such as DOPA is moderate in many cases, with assistance from caregivers often required as an intervention [33]. Accordingly, for long-term monitoring, a minimal-form-factor passive device would seem to represent the ideal compromise, and the positive results obtained with consumer-grade ring devices should be grounds for future development. This is backed by multiple reports demonstrating patient adherence to digital monitoring technologies waning over time, with typical reductions of up to 53% within 6 months in one study [34], dropping to less than 40% in other studies [35], and even in best-case scenarios, 70% adherence at 12 months is regarded as a benchmark [36]. In reality, though laudable, when considering that the disease trajectory can span 40+ years in total, it is appropriate to consider context and the reality that patient (consumer) burden needs very careful consideration (Figure 1). In the case of smart rings, any accuracy–adherence trade-offs would be compensated by long-term statistical benefits derived from detecting within-person changes and reducing measurement noise through enhanced sampling frequencies. Indeed, a critique of the Stanford sleep laboratory study likened the utility of smart-ring-derived measures akin to bathroom scales—ubiquitous, easy to use, but less accurate than sophisticated, calibrated devices, able to alert the user to sudden changes [24].

## 4. Integrating with Managed Care

Following diagnosis and staging, PD patients undergo periodic assessment typically performed by attending neurologists against scales such as MDS-UPDRS and Hoehn–Yahr [2,3]. A critical component of care involves detection and management of progression through stages and the associated need for escalation in care, symptomatic medications and eligibility to enroll in clinical trials. As such, integrating the outputs from a passive monitoring device into the patients’ EHR and managed care plan would be critical. Assuming the outputs of such included a daily electronic sleep diary uploaded direct to cloud, the HCP could monitor any changes between scheduled visits and also be alerted to specific high-risk anomalies. These could include the likelihood of falls related to chronic sleep anomalies or hallucinatory events detected by combinations of sleep and cardio-derived measurements. The latter could signal a transition from mid- to later-stage disease progression [37], and baseline data could be useful to inform the potential need to alter symptomatic medication dosing or prompt discussions on lifestyle interventions including dietary and physical activity regimens. Collectively, this could help improve many components of care and QoL, including medication adherence, optimized sleep counseling, scheduling of clinical visits and screening intervals. More critically, such an approach may help detect early symptoms associated with the prodromal stages, triggering early intervention and formal screening. An example is in idiopathic RBD, which can produce signals well before any motor symptoms present [38], and is becoming firmly established as a prodromal marker for α-synucleinopathy, with phenoconversion rates of 7–9% annually reported [39]. The benefits of this are myriad, including identifying critical cohorts of patients who may be candidates for disease-modifying clinical trials, where it can be reasoned that the chances of success are highest in the early stages of disease (Figure 2). Given that the prodromal phase can stretch over two decades and formal stages last an equal duration, it would seem imperative that any monitoring technology be at the lowest patient burden level possible (Figure 2). This might also prove appealing to individuals who are deemed at-risk but presently devoid of symptoms (e.g., familial history, identified omic risk factors associated with RBD). Of significance, progress using wearable devices has already demonstrated efficacy in identifying iRBD, and concerted effort in high-adherent (ring-based) devices would thus seem warranted [39].

## 5. Compromising and Optimizing Device Capabilities

As articulated in Figure 1, we believe the smart ring offers a near-ideal compromise for data gathering based on minimal friction for patients and the capacity to capture wide-ranging signals. Although primary interest is focused on a limited battery of sleep-derived metrics, there are a number of variables which could be considered in the design, roll-out, and eventual evolution of such a device. Among these are power sources which could involve a cable-based or contact-free recharging cell. Other options could be kinetic motion charging cells which would remain on the finger permanently, or a hybrid solar-assisted cell. The requirements for such are intricately linked to power drain on the device, which in turn is influenced by the number of powered features and its capabilities, for example, direct to cloud or Bluetooth connectivity for data upload/download. Assuming these operations (data capture, data transmission) are all conducted passively, the only patient interaction would be the device charging requirement, suggesting this should be a priority to minimize, particularly given the possible multi-decade lifespan of the device and its deployment (Figure 2). As is the case with existing smart devices, it is reasonable to expect that technological advances in sensing and associated algorithms, device charging procedures, and data transmission protocols could all be upgraded by direct-to-device firmware upgrades periodically, minimizing user burden. Additionally, data security provisions, which vary from region to region and are continually amended in the case of personal health data, will need to be upgraded, or when a user moves from one region to another temporarily or more permanently. A key to success in this aspect will be to establish a defined standard for data, and limit the commercial bias and influences on data sovereignty, particularly relevant given the lifespan of the device (Figure 3). In this regard, open-source approaches may be highly desirable.

## 6. Tracking Additional Measures

The simplest and arguably least controvertible single measure to capture is overall sleep, coupled with any detectable anomalies of relevance to symptoms. However, the smart ring platform offers the opportunity to capture other signals, or to incorporate multiple measures from a single device, subject to constraints imposed by power drain (Figure 3). The following are some examples of potential features which have already been explored in isolation and thus offer promise for adaptation in a longitudinal monitoring device.

### 6.1. Heart Rate Variability (HRV)

The calculation of HRV is a common metric offered in a number of smart health tracking devices where millisecond-level changes between heart beats are correlated with stresses to the sympathetic and parasympathetic nervous systems. A high HRV signals resilience and positive cardiovascular fitness, whereas low variability is associated with stress, fatigue, and poor sleep and suggests a higher risk for heart disease. In the case of PD, studies have examined the impact of the disease on HRV in untreated patients [40]. Across a cohort of 50 patients and 55 healthy controls, disturbances in the autonomic nervous system were tracked through cardiovascular autonomic reflexes and HRV at rest. The PD group demonstrated attenuated HRV and blood pressure anomalies, and, among patients with known hypokinesia/rigidity, demonstrated greater HRV deficit than those with early onset tremor, suggesting such a marker could be useful in tracking progression (Figure 3). Such measures would be easy to introduce as one of the leading smart ring platforms, the Oura ring, routinely provides users with this feature. Under standard conditions, HRV is monitored exclusively during sleep cycles across continuous 5 min sampling periods, presenting a mean score (measured in milliseconds) the following morning. Users are able to access both their average and maximum HRV and view trends and projections which can be interpolated with key diary features. Though the latter requires active user engagement, it is conceivable that the data could be instead transmitted directly to a caregiver, proxy or HCP to eliminate friction on the subject. As with any experimental/emerging tool, deployment at scale will help in refinement and to identify and overcome limitations inherent to disease heterogeneity, which is a particular feature of PD, with distinct sub-phenotypes identified [41].

### 6.2. PD-Related Tremors

Aside from sleep-derived metrics, one of the key symptoms of the disease is the occurrence and progressive worsening of hand tremors. Although accurate measurement of this typically involves sophisticated laboratory techniques, given that the smart ring would be residing on one of the eight fingers of the hand, it is logical to assess if functionality could be added to track this key metric. Some relevant work has been conducted using a Leap Motion device, which monitored hand movements of 55 patients across all 25 joints including the fingers, thumb, and wrist [18]. The degree of movement was captured in terms of volume, velocity and acceleration and revealed that the ends of fingers exhibited the greatest velocities and accelerations, with those on the right side showing greater effects than the left hand. Significantly, the correlation of motion scores with clinical MDS-UPDRS scores was demonstrated, suggesting utility in longitudinal tracking [18]. In another case, the performance of a custom smart ring device containing both a 3-axis gyroscope and accelerometer to detect acceleration and the angular velocity of fingers was studied [22]. The device was capable of detecting tremor signals with high accuracy and the prototype also incorporated Bluetooth Low Energy (BLE) capabilities, enabling tremor data to be transferred to an external device in real time with low energy demand [22]. In a similar vein, a smart ring for detection of rest tremors has been reported. The device, known as an RT-ring, captures inertial tremor data and deploys machine learning capabilities to estimate resting tremor patterns [21]. A total of 67 subjects were studied including 42 with known dopaminergic deficit and 25 controls. Among the symptomatic group, the ring showed 85.0% sensitivity, 90.9% specificity, and 87.9% balanced accuracy in predicting tremor-related imaging outcomes (^123^I-ioflupane DaTscan), showing promise as a classification tool in PD [21]. The authors also note the utility of this in low-income countries, where access to standard classification approaches (nuclear imaging infrastructure) is currently very limited.

### 6.3. Detecting on and off States

One of the disease hallmarks for PD patients taking symptomatic therapies (dopamine class) are so-called “on” and “off” states. During on states, movement and related symptoms are well controlled, allowing the patient to function with relative ease, whereas in off states (emblematic of when therapies have worn off), symptoms worsen until dosing is readministered. The fluctuations occur more frequently as the disease progresses, with the brain losing capacity to store natural dopamine, requiring frequent re-dosing of medications to prevent sudden declines in mobility. The ability to detect approaching off states would be of benefit to patients and caregivers alike in order to prevent the potential for adverse events and maximize quality of life. Although the deployment of smart ring devices to classify on/off states directly has yet to be demonstrated, a considerable body of work has been reported on the use of machine learning methods applied to motor function data which are relevant. In one study, range motion data derived from gait and shank were subjected to 12 machine learning models, allowing refinement of the classification algorithm [19]. Ultimately, the authors were able to successfully classify on/off states with a high degree of sensitivity and accuracy (AUC = 0.956; sensitivity = 0.8947, 95% CI: 0.6686–0.9870; accuracy = 0.8421, 95% CI: 0.6875–0.9398) [19]. Although motion-derived data would be expected to be limited from smart rings, there is promise that refined algorithms and newer technologies such as next-generation IMU sensors could become available to decipher key data from simple hand motions in the future, aiding in classification performance.

One clinical study directly assessed the impact of different monitoring approaches on classification [42]. This multicenter, single-blind, cluster-randomized trial evaluated the effectiveness of three approaches in clinical practice for 156 patients across 35 neurology centers. This included clinical visit data, Hauser diary entries, and Holter (STAT-ON^®^) reports [42]. A key finding was that the choice of motor fluctuation monitoring method did not significantly impact clinical classification, underscoring the need for better understanding of tools deployed in real-world settings. To this point, the potential for a passive (smart ring) technology to track signals un-interrupted longitudinally over long periods could be very useful in assessing the impact of on/off states under real-world conditions, and the impact on activities of daily living.

### 6.4. Assessing Functional Capacity

A number of interesting prototype rings have been reported for use in PD research and among these, the Tau ring is noteworthy [20]. The *τ*-Ring integrates PPG, IMU, TEMP, and NFC sensors and was designed around the open-source android platform to facilitate adoption. In addition to traditional measures including heart-rate monitoring, the IMU data channels are configured to assess ring-based handwriting and gesturing. This is significant as it could serve as a frictionless means to capture activities of daily living of patients, and the potential impact of tremor, off states and sleep anomalies over a sustained period. This could inform decisions to optimize medication regimens and other interventions over time. One such example could be switching from a daily dosed regimen of a dopaminergic agent to a controlled release version, which could minimize off states induced by patients’ lapses in medication adherence.

Adding any of the above features and capabilities to a smart ring designed around sleep monitoring would seem feasible but will likely come at the expense of power consumption, weight, and possibly the need for user interaction, which in turn introduces friction (Figure 1, Figure 2 and Figure 3). Our understanding of disease trajectory and etiology will likely improve over the coming years, as will the performance and characteristics of battery technologies, digital sensors, wireless systems and medical-grade algorithms. Accordingly, it may become viable or desirable to develop multi-channel sensors, bolstered by new technologies or emerging needs from the healthcare system, patients, and caregivers. In the longer term, one hopes that therapies emerge to treat, reverse, or prevent the disease—rendering such technologies obsolete.

## 7. Challenges and Priorities

The potential benefits of deploying smart-ring technology for longitudinal tracking in PD are myriad. However, implementation will require considerable attention to a number of key details surrounding the algorithms used to process captured data, device firmware and the sensors themselves. First and foremost, it will be pivotal to optimize for signal accuracy, specificity and selectivity relative to sleep disturbances among subjects. These are by definition composite measures, impacted by diverse elements including motor symptoms, circadian disruption, sleep apnea, autonomic dysfunction, and restless leg syndrome, and likely to be influenced by comorbid polypharmacy effects (e.g., antidepressants, beta-blockers), symptomatic (e.g., dopaminergic) therapies, and potentially disease-modifying therapies. This will demand careful interpretation by attending clinicians or neurologists, to ensure actionable insights are attained. For example, detection of hallucinatory events, disease progression markers or medication effects will require precision medicine approaches to be adopted; thus, it will be critically important to engage the healthcare community in the design of the workflows associated with the device to ensure integration into the subjects’ EHR is approached appropriately. Linked to this, as actionable events are detected, the device will likely need to be classified as a medical device, and thus require appropriate regulatory and data privacy approvals and qualifications. Lastly, given the extended timeframes anticipated for the devices, it will be important to consider continual evolution of both the algorithms and devices themselves, to ensure version tracking, calibration, and comparability are consistent to minimize data gaps and maximize the utility of captured data longitudinally. Learnings from the smartphone and smart watch industries will be insightful and it is likely that common metrics and data standards will evolve to harmonize methods for data capture, storage and integration in health records. Addressing these challenges will benefit from early and continual dialog between technology developers, regulatory bodies, the healthcare community and patient advocacy groups.

## 8. Expanding to Other Therapeutic Areas

Although the primary motivation for this perspective was PD given the known impact of the disease on both sleep quality and its idiosyncrasies, the approaches described herein could be of benefit to a number of other neurodegenerative diseases. These include Alzheimer’s disease and related dementias, where decreased deep sleep, fragmented sleep and sundowning are known features, and Huntington’s disease, where dysfunction of the basal ganglia results in involuntary movements which cause poor sleep efficiency and fragmented sleep.

Likewise, a number of neuromuscular diseases including ALS and myotonic dystrophy result in nocturnal hypoventilation, resulting in frequent awakenings during the sleep cycle. Other examples include multiple sclerosis, associated with insomnia; epilepsy where nocturnal seizures can disrupt sleep architectures; restless leg syndrome where restorative sleep is severely impaired; and stroke/TBI where disrupted circadian rhythms, insomnia, and hypersomnia are common.

## 9. Conclusions and Next Steps

There is evident potential for the large-scale deployment of smart ring technologies for the care of PD patients through sleep and symptom monitoring. For this to become adopted and integrated into managed care, however, will require progress and dialog at the intersection of health technology innovators, healthcare providers, patient support groups and medical professional societies. In the case of technology, in addition to optimizing algorithm architectures, solving for direct-to-cloud data transmission, and extending battery life, decisions on which health-related features and how to monitor them need to be addressed, including dashboard-style integration of data into patient EHRs and correlation with current scoring systems such as MDS-UPDRS. Motivation for this could also be enhanced by thorough examination of health economic outcomes (HEOR) and the potential impact on actuarial scales such as disability-adjusted life years (DALYs), providing incentives for payers to stimulate adoption of the technologies [43]. None of this would be meaningful, however, without patient engagement at the earliest stages. This should include field testing technologies to optimize the human–device interface, and determining the preferred level of patient engagement including access to and sovereignty of derived datasets.

For all the above, we advocate

1. Increasing awareness for the technologies in scholarly venues such as medical conferences and journals such as this.
2. Facilitating dedicated stakeholder workshops including patient groups and developers.
3. Actively engaging professional societies, regulatory bodies and facilitators such as the Critical Path Institute to amplify impact in related areas [44].

Beyond PD, neurodegenerative diseases afflict over 50M patients globally, and the solutions outlined herein could be broadly applicable [45]. Although much needs to be done to fully realize the vision outlined herein, there is considerable optimism that the challenges are addressable. First and foremost, health-related sensors and the technologies they rely upon are advancing at an extremely rapid pace. Driven by the consumer industry, we are in the midst of a revolution where adoption of digital devices for health tracking has moved beyond the technophile community into mainstream society [46]. This has a multi-fold impact on adoption spread by lowering the barrier to entry through affordability [more competition, economies of scale] and normalizing the use of devices across multiple demographics. The latter is of high importance to patients who previously may have had to contemplate the use of obtrusive, high-friction devices, which run the risk of drawing unwanted attention to them. Consumer devices such as the iPhone, Fitbit and Oura Ring are seen more as fashion accessories than health-monitoring devices, and given the intended deployment (over multiple decades), this becomes critical. It is perhaps difficult to predict the changes we will see in the form factors and capabilities of these devices over the next 50 years; however, one constant to reflect upon is that the dimensions of the human finger are likely to stay within current ranges. It is thus reasonable that a minimal-form-factor smart ring deployed today will not look out of place in 2076. Let us hope that by this time, parallel developments in drug development will render the debilitating diseases described herein as both curable and preventable, rendering the need for disease-monitoring technologies obsolete.

## Figures and Tables

**Figure 1:**
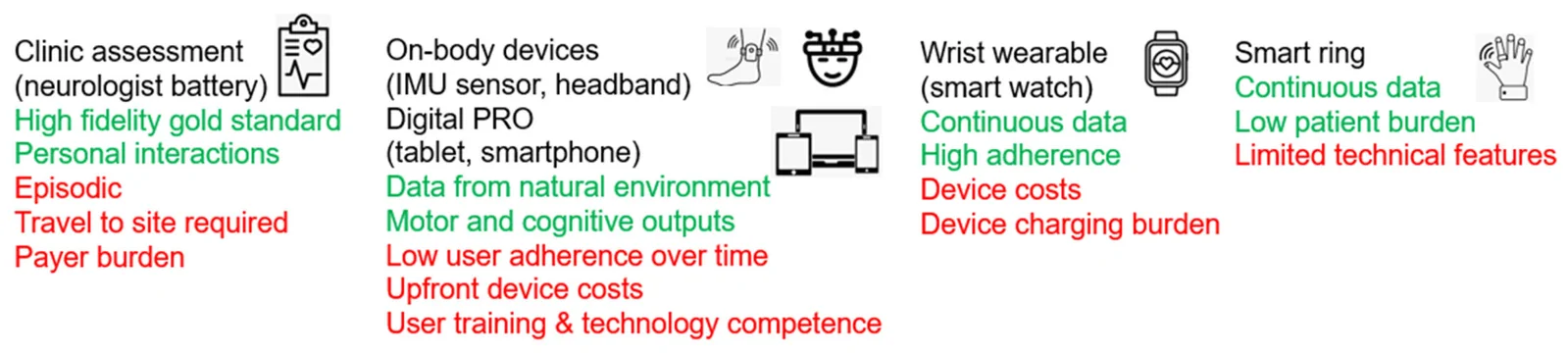
Estimated device burden and symptom tracking using DHTs in Parkinson’s disease. (red = limitations, green = benefits).

**Figure 2:**
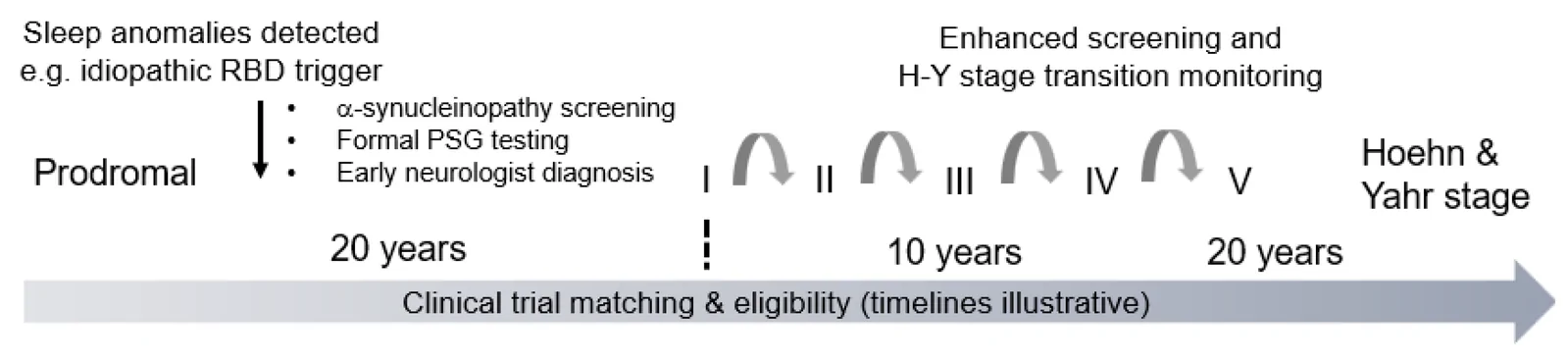
How longitudinal smart ring monitoring can form of the patient journey in PD.

**Figure 3:**
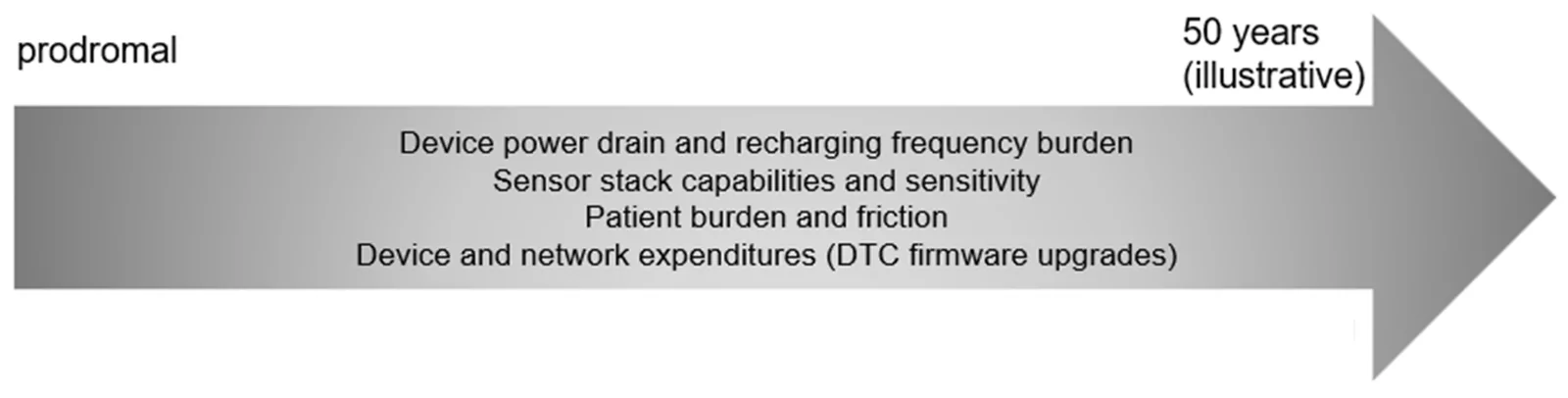
Optimizing smart ring features and capabilities for longitudinal monitoring.

**Table 1:** Sleep-related symptoms associated with Parkinson’s disease.

Circadian Rhythm Disruptions	Respiratory Anomalies	Nocturnal Hallucinations
REM Behavior Disorder (RBD)	Movement Disorders	Daytime Sleepiness
Nocturnal Immobilization	Chronic & Episodic Insomnia	Chronic & Episodic Fatigue

## Data Availability

Data associated with the manuscript is available from the authors.
